# Evaluation of a Genetic Test for Diagnose of Primary Hypolactasia in Northeast of Iran (Khorasan)

**Published:** 2012

**Authors:** Maryam Alizadeh, Ariane Sadr-Nabavi

**Affiliations:** 1 *Departmant of Human Genetic, Mashhad University of Medical Sciences, Mashhad, Iran*; 2 *Iranian Academic Centres for Education, Culture and Research (ACECR) *

**Keywords:** Hypolactasia, Iranian Population, LPH

## Abstract

**Objective(s):**

Primary or adult type hypolactasia, the most common enzyme deficiency in the world, is due to reduced lactase activity in the intestinal cell after weaning. Lactase non-persistence is inherited as an autosomal recessive trait. A DNA variant, single nucleotide polymorphism C/T−13910 which is located on 13910 base pairs (bp) upstream of the lactase gene (LCT) at chromosome 2 has been show to associate with the lactase persistence/non-persistence. The prevalence of the C/T-13910 variant is different for hypolactasia in European, Asian, African-American and Northern African populations. In this study, we investigated, for the first time the allele frequent of the single nucleotide polymorphism C/T−13910 in the Iranian population in khorasan province with hypolactasia.

**Materials and Methods:**

Peripheral blood was collected from 100 subjecs with primary hypolactasia and 100 healthy individuals as a control group. Genomic DNA was extracted. The genotype was analyzed with the PCR-RFLP method. A statistical analysis was performed by chi-square test using SPSS software. A *P*-value of <0.05 was considered statistically significant.

**Results:**

In case group allelic frequency for SNP T-13910C (C, T) was respectively 95%, 5% vs. control group 86% and 14%. Genotype frequency (CC, CT, TT) in patient group was 90%, 10%, 0% vs. control group 74%, 24% and 2%. So according to our findings, there were significant differences between allelic frequencies (*P*=0.03), and in genotype frequency between case and control groups (*P*=0.006).

**Conclusion:**

Based on our results, analysis of C\T-13910 polymorphism can be used as a simple genetic test for diagnosis of primary type hypolactasia in the Iranian population.

## Introduction

Lactose disaccharide is the main carbohydrate in mammalian milk that is comprised of glucose and a galactose molecule. Lactase or lactase-phlorizin hydrolase is a brush border membrane enzyme in the intestinal epithelial cells that catalyzes lactose (1, 2). Primary or adult type hypolactasia, the most common enzyme deficiency in the world, is due to reduced lactase activity in the intestinal cell after weaning (3).

During childhood, lactase activity reduces to about 10-15% of its activity at birth. This condition is genetically programmed and irreversible reduction of lactase occurs in more than 75% of the world’s population (4-6).

In some population, mainly in people of north European descent, lactase activity continues post weaning and into adulthood. Lactose intolerance occurs when the presence of undigested lactose in the colonic lumen causes gastrointestinal symptoms such as abdominal pain, bloating, flatulence, and diarrhea (7, 8). Kruse *et al* (1988) showed that the LCT gene is located on chromosome 2 (9). In 1973 Sahi et al, concluded that hypolactasia is controlled by an autosomal recessive single gene (10). 

Enattah *et al* (2002) analyzed the region flanking the LCT gene at 2q21. They found that C/T polymorphismtat which is located on 13,910 base pairs upstream of the LCT gene, in intron 13 of the MCM6 gene; have complete association with lactase persistence/non-persistence trait (11). 

In vitro studies demonstrate that T-13910 allel is responsible for increased LCT promoter activity. -13910 regions contain a strong enhancer so that the T-13910 allel enhances the LPH promoter 4 times more than the C-13910 variant (12). Analyses of intestinal biopsies have demonstrated that the C/C-13910 genotype is associated with low lactase activity (13). The use of the C/T-13910 variant can be a robust marker for adult-type hypolactasia, however, in some sub- Saharan African groups, C/T-13910 variant cannot serve as a predictor for lactase persistence (14). 

Studies have shown that there is a high prevalence of lactose malabsorbtion among Iranians. The aim of this study was to validate a genetic test for adult type hypolactasia in an Iranian population.

## Material and Methods

***Subjects***

Peripheral blood was collected from 100 subjects with primary hypolactasia (36 male, 64 female with the mean age of 31±18/1 years) and 100 healthy individuals as a control group (46 male, 56 female with the mean age of 45±16/1 years). Informed consents were obtained from all participants. The exclusion criteria were disease associated with secondary hypolactasia such as celiac, severe gastroenteritis, crohns disease, and ulcerative colitis.

***Genetic test***

Genomic DNA was extracted from leukocytes collected in EDTA tubes (5PRIME kit GmbH, D-22767 Hamburg). The previously described primers, LAC-C-M-U: 5′-GCTGGCAATACAGATAAGATAATGGA-3′ (position 26611–26636) and LAC-C-L-2: 5′-CTGCTTTGGTTGAAGCGAAGAT-3′ (position 26790–26811, Accession number: AY220757) was used to amplify the region flanking the C/T13910 polymorphism (12). Hundred ng of genomic DNA was used as template in a reaction volume of 50 μL, containing 36.4 μl ddH2O, 5 μl 10X reaction buffer, 25 mM MgCl2, and 10 mM dNTP, 10 pmol of each primer and 5U of Taq DNA polymerase (Genet Bio). Cycling conditions were 94°C for 5 min, followed by 30 cycles of 40 sec at 94°C, 40 sec at 58°C and 40 sec at 72°C, and 5 min at 72°C as the final extension step. Mulcare et al (13) introduced a base change in the penultimate base of the LAC-C-M-U primer (G instead of T) so that the PCR product could be digested by HinfI as the T allele exists. PCR products (10 μl) were digested with restriction endonuclease HinfI (0.25 U) incubated at 37°C overnight (the final volume 20 μl), followed by enzyme deactivation at 65°C for 20 min. HinfI digestion of PCR products with the T allele resulted in 177 bp and 24 bp fragments; digestion of the C allele generated a single fragment of 201 bp.

**Figure 1:**
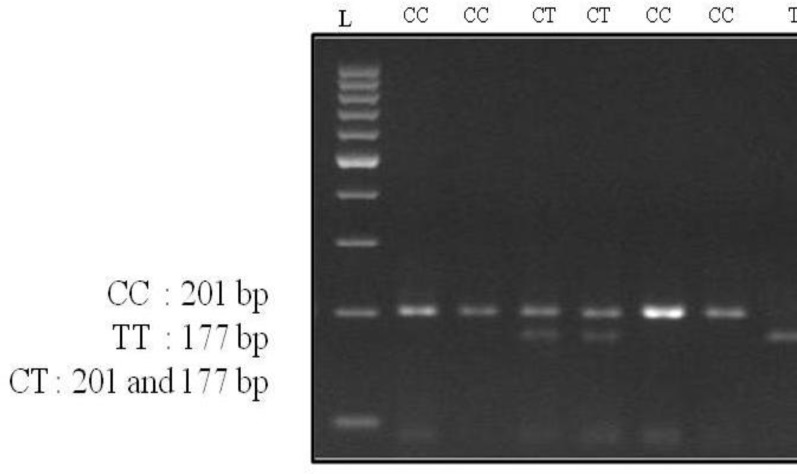
Agarose gel (2.5%) electrophoresis of HinfI digested PCR products. 100 bp DNA ladder (Fermentas) was used as marker of DNA fragment sizes. The CC genotype was confirmed by a single band of 201 bp, the TT genotype indicated by a single band of 177 bp, and; CT genotype by two bands of 201 bp and 177 bp

Positive controls were included to confirm complete digestion. Digested PCR products were observed on a 3% agarose gel stained by ethidium bromide. Samples showing a single band of 201 bp were typed as the CC genotype and a single band of 177 bp as the TT genotype; two bands of 201 bp and 177 bp represented the CT genotype (Figure 1). The CT and TT genotypes associated more with lactase persistence trait previously and the CC genotype represents hypolactasia (14, 15).

***Statistical analysis***

Statistical analysis was performed by chi-square test using SPSS software. A P- value of <0.05 was considered statistically significant.

## Results

For case and control groups, tests of the validity of Hardy-Weinberg equilibrium showed no deviation from expectation. In the case group, allelic frequency for SNP T-13910C (C, T) was respectively 95% and 5% vs. control group 86%, 14% in genotype frequency (CC, CT, TT) in patient group was 90%, 10%, 0% vs. control group 74%, 24% and 2% displayed in Table 1.

Thus, according to our findings there were significant differences between allelic frequencies (*P*= 0.03), and in genotype frequency between case and control groups (*P*= 0.006).

**Table 1:** Distribution of C/T-13910 genotype in the subjects studied

Genotype	Case (100)	Control (100)
C/C C/T T/T	90% 10% 0%	74% 24% 2%

## Discussion

The prevalence of primary hypolactasia varries in the world's population, ranging from 2% in northern Europe to 100% in some Asian countries (5). In the present study, our data indicates that frequency of CC-13910 genotype (lactase non-persistence) is high in both patient and control groups, 90% and 74% respectively. The prevalence of the C/T-13910 polymorphism is consistent with the prevalence of adult type hypolactasia reported in two other studies in Iranian population (15, 16). Using lactose tolerance test, Sadre *et al* indicated that there is a high prevalence of lactose malabsorbtion and lactose intolerance among Iranians (15). 68% of their study subjects were lactose malabsorbers. They also indicated that there was high rate of milk rejection in Iranian school age children (15).

Rahgozar et al studied C/T variance associated with primary hypolactasia in a rural population in the central of Iran. They showed that the prevalence of primary hypolactasia was 84% in their study population. In Caucasian origin population, lactase persistence trait is relatively common. Iranian population is primarily of Caucasian origin but contrary to them, the present data showed for the first time that variances underlying lactase persistence, T-13910, is rare in Iranian population with a frequency of 14% in healthy subjects. This fact could be explained by tradition of consanguineous marriages that is high in Iranian population.

Due to this condition, frequency of recessive allele, C-13910 allele, becomes very high in comparison with dominant allele. However, it is possible that there are additional mutations that have not been discovered yet. However, frequency of C allele that is associated with lactose intolerance was significantly higher in patient group compared with control group (*P*= 0.03). There was significant difference in CC genotype between patient and control groups (*P*= 0.006). Based on our results, analysis of C\T-13910 polymorphism can be used as a simple genetic test and a non-invasive test for diagnosis of primary adult type hypolactasia. In this study we showed for the first time that genetic test could be used with high sensitivity, time and cost effectiveness for hypolactasia in Iran.

## Conclusions

In this work we showed for the first time that, there is association between CT-13910 polymorphism and hypolactasia in the Khorasan population. Analysis of the C/T-13910 variant can be a very specific and sensitive test for predicting hypolactasia in a patient population with suspected lactose malabsorption. 
